# Combined scleral suture-fixated intraocular lens implantation and Iris cerclage pupilloplasty for aphakic eyes with traumatic mydriasis

**DOI:** 10.3389/fmed.2026.1958055

**Published:** 2026-09-29

**Authors:** Mengxiao Wu, Hongfei Ye, Ping Fei, Jie Peng, Jiao Lyu, Yu Xu, Peiquan Zhao

**Affiliations:** Department of Ophthalmology, Xinhua Hospital, Shanghai Jiaotong University School of Medicine, Shanghai, China

**Keywords:** aphakia, intraocular lens implantation, iris cerclage, pupilloplasty, scleral fixation, traumatic mydriasis

## Abstract

**Purpose:**

The aim of this study was to present the observational outcomes of scleral suture-fixated intraocular lens (IOL) implantation combined with iris cerclage pupilloplasty to treat aphakic eyes with traumatic mydriasis.

**Materials and methods:**

A retrospective review was performed consisting of 9 aphakic eyes in 9 patients with traumatic mydriasis who underwent scleral suture-fixated IOL implantation combined with iris cerclage pupilloplasty.

**Results:**

The mean age was 18.9 ± 18.1 years and the mean follow-up time was 6.3 ± 6.1 months. Best-corrected visual acuity improved from 0.94 ± 0.75 logarithm of the minimum angle of resolution (logMAR) to 0.75 ± 0.67 logMAR after the surgery (*p* = 0.042). Intraocular pressure decreased from 17.81 ± 6.37 mmHg to 13.70 ± 3.64 mmHg (*p* = 0.046). The pupil diameter reduced from 8.851 ± 0.686 mm to 5.263 ± 1.361 mm (*p* = 0.0001). In addition, the symptom of photophobia had significantly alleviated. The difference of endothelial cell density was not statistically significant postoperatively (*p* = 0.698).

**Conclusions:**

Scleral suture-fixated IOL implantation combined with iris cerclage is an effective method to achieve functional and cosmetic outcomes for aphakic eyes with traumatic mydriasis.

## 1 Introduction

Iris-lens composite defect represents a common presentation of anterior segment disruption following ocular trauma (1). In the management of certain severe cases, primary lens extraction and vitrectomy without intraocular lens (IOL) implantation may serve as a safe therapeutic strategy. Among patients requiring secondary operation, aphakic eyes accompanied by traumatic mydriasis and inadequate capsular support pose substantial clinical challenges (2). Traumatic mydriasis constitutes a severe form of iris dysfunction, leading to blurred vision, disabling glare, halos, photophobia, and compromised cosmetic appearance (3). Accordingly, an appropriate surgical approach is critical for these patients to achieve favorable anatomical and functional outcomes.

In patients with pathological mydriasis and extensive iris defects, anterior chamber IOL implantation carries a spectrum of complications, including corneal decompensation, glaucoma, hyphema, recurrent iritis, and cystoid macular edema (4, 5). Compared to anterior chamber IOLs, scleral-fixated posterior chamber IOLs (PCIOLs) enable IOL positioning in the physiologic anatomic location and mitigate complications in eyes with deficient capsular support (6). Scleral fixation can be broadly categorized into suture-based and sutureless approaches, with the selection determined by the individual patient's clinical conditions. The Yamane technique streamlines surgical maneuvers and expedites the surgery procedure, is currently the most popular sutureless intrascleral IOL fixation (7). However, this approach may not be ideal for pediatric patients, individuals with high myopia, or those presenting with scleral scarring secondary to pars plana vitrectomy incisions. Under such circumstances, the IOL haptics are liable to shift, which may elevate the risk of lens dislocation. Accordingly, scleral-sutured IOL fixation can be a more favorable alternative in these scenarios. In eyes with permanent mydriasis, the iris cerclage technique has been successfully applied to reconstruct a round pupillary aperture, achieving satisfactory functional and cosmetic results (8–10).

Though iris reconstruction combined with iris-claw IOL implantations and Yamane technique have been reported in the literature, the complications and long-term stability remain subjects of concern (2, 8, 9). Scleral suture-fixated IOL implantation combined with iris cerclage pupilloplasty represents an effective procedure for restoring a nearly normal anterior segment structure, however, relevant clinical reports remain limited. In the present study, we first report the clinical outcomes of the combined technique for the management of aphakic eyes with traumatic mydriasis.

## 2 Material and methods

A retrospective review was performed of aphakic eyes with traumatic mydriasis that underwent scleral suture-fixated IOL implantation using either a single-piece PCIOL (RayOne RAO600C; Rayner Intraocular Lenses Limited, Worthing, West Sussex, United Kingdom) or a three-piece PCIOL (Tecnis ZA9003; Johnson & Johnson, Santa Ana, California, USA; or MA60AC; Alcon Laboratories, Fort Worth, Texas, USA) combined with iris cerclage pupilloplasty between April 2020 and April 2026. This study was approved by the Institutional Ethics Committee and informed consent was obtained from all patients. Inclusion criteria were as follows: (1) traumatic mydriasis; (2) previous lens extraction surgery due to trauma-induced conditions including cataract; (3) lens dislocation; and (4) retinal detachment. Exclusion criteria were as follows: (1) severe ocular surface disease (e.g., severe dry eye or keratitis); (2) previous iris surgery; (3) uncontrolled systemic diseases (such as uncontrolled diabetes mellitus or hypertension, which could affect surgical safety and wound healing); (4) congenital iris-lens defects; and (5) a follow-up less than 3 months. Medical records including gender, age, surgical indications, ophthalmic comorbidities, IOL implantation procedures and postoperative complications were collected. All patients underwent ophthalmic evaluations including best-corrected visual acuity (BCVA), intraocular pressure (IOP), slit-lamp biomicroscopy, degree of subjective photophobia, endothelial cell density (ECD), optical coherence tomography (OCT) preoperatively and at the last visit postoperatively. Pupil diameter was measured using intraoperative surgical microscope images under standardized illumination and magnification. All measurements were performed under photopic conditions, as the operating microscope was set to a fixed illumination intensity during image acquisition. Preoperative pupil diameter was recorded at the beginning of the surgery before any intraocular manipulation, and postoperative pupil diameter was measured at the end of the surgery after IOL implantation and iris cerclage pupilloplasty were completed, with the anterior chamber fully formed and intraocular pressure normalized. Both preoperative and postoperative measurements were obtained from images captured under identical illumination settings. Pupil diameter for each eye was calculated as the average of the horizontal and vertical diameters, and calibration and analysis were performed using ImageJ software (National Institutes of Health, Bethesda, Maryland, USA).

### 2.1 Scleral suture-fixated IOL implantation

For scleral suture fixation of single-piece IOL, the detailed surgical procedure is as follows. Two scleral pockets are formed posteriorly from the limbus at 3 o'clock and 9 o'clock positions utilizing a crescent blade. Dissection was carried out perpendicular to the limbus, extending 3.0 mm while maintaining a uniform scleral depth of 300*μ*m. Subsequently, the superior corneal incision and side paracenteses are performed (Figure 1A). Two puncture sites are delineated using calipers at the midpoint of the scleral pockets, positioned 2.0 mm posterior to the limbus on both sides. The straight needle of a 9-0 polypropylene suture (Mani, Tochigi, Tokyo, Japan) is introduced at one puncture point at 3 o'clock through the conjunctiva and full thickness of the pocket, threading through the anterior chamber and docked into the opening of a 30-G needle which is introduced at 1.0–2.0 mm adjacent to the puncture point at 9 o'clock and externalized (Figure 1B). Identical maneuvers are executed at the 9 o'clock puncture site, resulting in the formation of a suture loop (Figure 1C). Subsequently, the paired sutures are externalized from the anterior chamber through the superior incision using forceps and cut into two halves, yielding two double-sutured ends for IOL fixation (Figure 1D). The leading haptic of the PCIOL is pushed out of the cartridge and tied with one end of the double sutures at the haptic end (Figure 1E). Following the injection of the leading haptic and the folded IOL optic into the anterior chamber, the trailing haptic is secured to the remaining suture end and subsequently introduced into the eye (Figure 1F). The needles attached to the suture ends passed at the 3 o’clock position is removed, and the suture loop at the 9 o'clock position is cut into two single suture ends. Each suture end is externalized through the scleral pocket opening using a Sinskey hook to grasp and withdraw the trailing suture (Figure 1G). The pairs of sutures at each scleral pocket are tied with tension-adjustable knots to achieve centralization of the IOL optic (Figure 1H).

**Figure 1:**
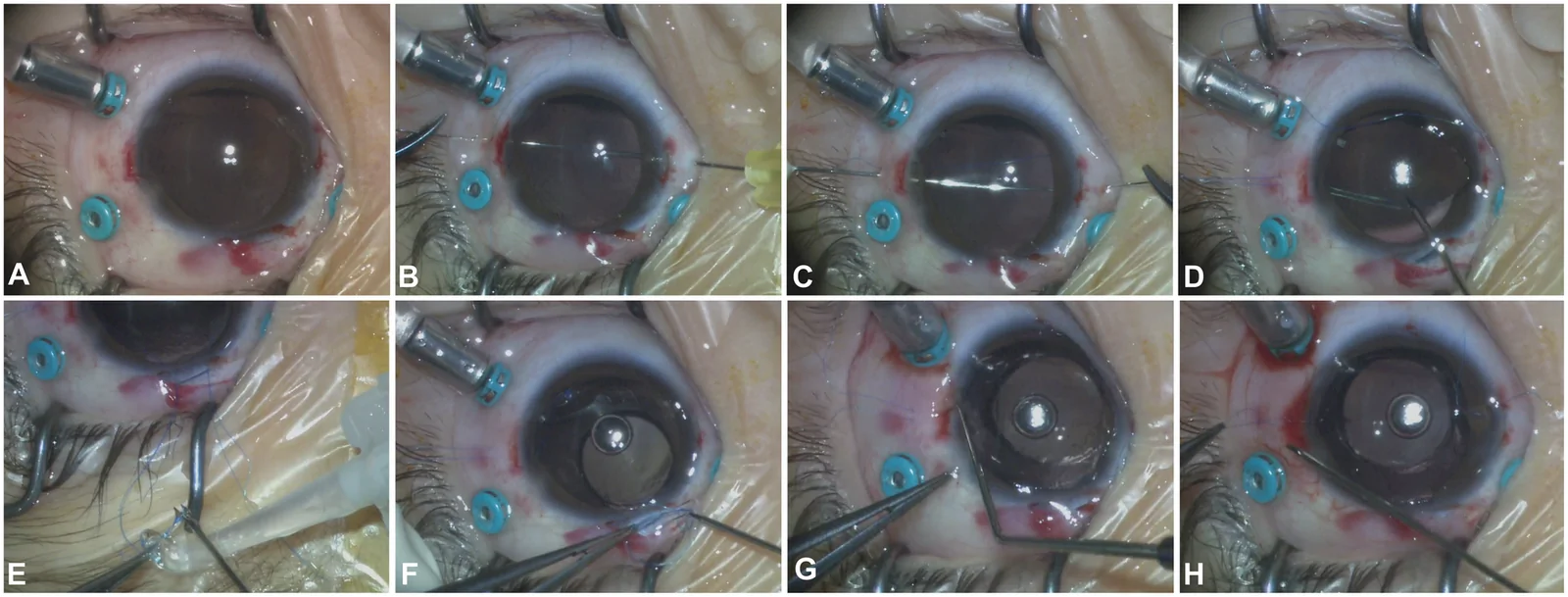
**(A)** Two scleral pockets are dissected using a crescent blade posteriorly from the limbus at 3 o’clock and 9 o’clock, the superior corneal incision and side paracenteses are performed. **(B)** After the puncture points are marked at the middle line of the pocket beds 2.0 mm posterior to the limbus on both sides, the straight needle of a 9-0 polypropylene suture is introduced at the puncture point at 3 o’clock, passing through the anterior chamber and docked into the opening of a 30-G needle which is introduced at 1.0-2.0 mm adjacent to the puncture point at 9 o’clock and externalized. **(C)** The identical maneuver is repeated at the other puncture site at 9 o'clock. **(D)** The pairs of sutures are retrieved externally through the superior incision and divided into two halves. **(E)** The leading haptic of the IOL is pushed out of the cartridge and tied with one end of the double sutures at the haptic end. **(F)** After the leading haptic and the IOL optic are injected into the anterior chamber, the trailing haptic is tied with the other end of sutures and subsequently inserted into the eye. **(G)** Each suture end is externalized through the scleral pocket opening by a Sinskey hook. **(H)** The pairs of sutures at each scleral pocket are tied with tension-adjustable knots.

For specific scleral suture fixation procedures of three-piece IOL, refer to the relevant literature previously published by our team (11).

### 2.2 Iris cerclage pupilloplasty

The superior corneal incision at 12 o’clock are already made for IOL implantation and the side paracenteses are made at 2, 4, 6, 8, 10 o'clock for iris cerclage. The iris adhesions were released by the 23-G vitreoretinal forceps. The forceps introduced through paracentesis at 4 o'clock is used to gently grasp the iris. The straight needle of 9–0 polypropylene suture (Mani, Tochigi, Tokyo, Japan) is introduced through the puncture point at 6 o'clock and passed through the iris approximately 1 mm from the pupillary margin. The needle is then passed through the pupillary margin to the iris surface at intervals of approximately 2 mm. At least 2 iris bites are taken between the puncture points at 6 o'clock and 4 o'clock. With the help of 23-G forceps, the needle is guided out via the side paracentesis (Figure 2A). The needle is re-introduced through the paracentesis at 4 o'clock and the same manipulations are performed between 4 and 2 o'clock (Figure 2B). The procedures are repeated between 2 and 12 o'clock (Figure 2C), 12 and 10 o'clock (Figure 2D), 10 and 8 o'clock (Figure 2E), finally 8 and 6 o'clock (Figure 2F). Until 360 degrees suturing of the iris is completed, both ends of the suture are tied outside the paracentesis at the 6 o'clock position and the pupil diameter is carefully adjusted through the suture tension (Figure 2G). Upon completion of the procedure, the superior incision is sutured, achieving a watertight seal at the side paracenteses (Figure 2H).

**Figure 2:**
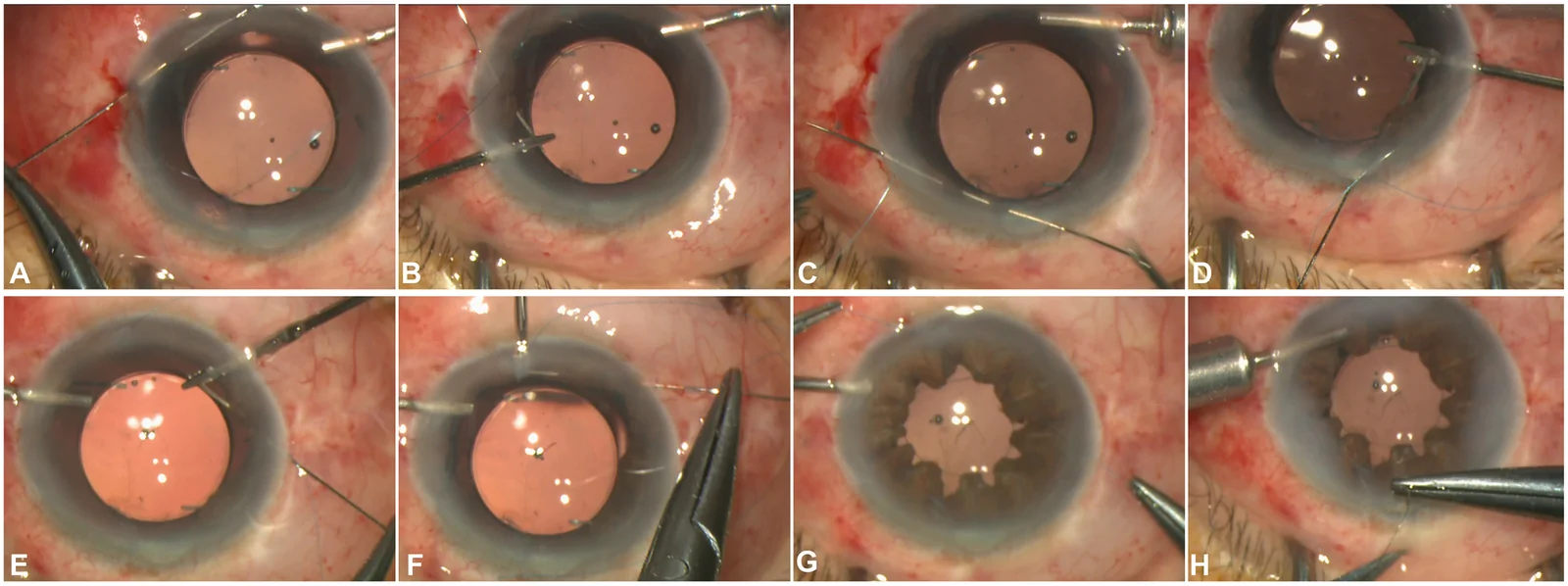
**(A)** the side paracenteses are made at 2, 4, 6, 8, 10 o’clock and the iris adhesions were released. The 23-G vitreoretinal forceps introduced through paracentesis at 4 o’clock is used to gently grasp the iris. The straight needle of 9-0 polypropylene suture is introduced through the puncture point at 6 o’clock and passed through the iris approximately 1 mm from the pupillary margin. The needle is then passed through the iris near the pupillary margin, taking bites at 2-mm intervals. At least 2 iris bites are taken between the puncture points at 6 o’clock and 4 o’clock. The needle is guided out via the side paracentesis at 4 o’clock. **(B)** The needle is re-introduced through the paracentesis at 4 o’clock and the same manipulations are performed between 4 and 2 o’clock. **(C–F)** The procedure is repeated between 2 and 12 o’clock, 12 and 10 o’clock, 10 and 8 o’clock, finally 8 and 6 o’clock. **(G)** Both ends of the suture are tied outside the paracentesis at the 6 o’clock position and the pupil diameter is carefully adjusted through the suture tension. **(H)** The superior incision is sutured and the side paracenteses are watertight.

All surgeries were performed by a single experienced surgeon (PQ.Z). Supplementary Video S1 shows one-piece IOL implantation with pupil cerclage suture, and Supplementary Video S2 shows the three-piece counterpart.

### 2.3 Statistical analyses

All analyses were performed by SPSS software version 26.0 for Windows (SPSS, Chicago, Illinois, USA). For descriptive and analytic purposes, the Snellen visual acuity was converted to the logarithm of the minimum angle of resolution (logMAR) units. To maintain analytical validity, we performed pairwise deletion for missing values in all paired comparisons, meaning that only complete data pairs were included for each specific test. Accordingly, the effective sample size varied slightly across different outcome measures, as specified in the corresponding tables. The differences between preoperative and postoperative BCVA were analyzed by Wilcoxon signed-rank test. The differences of preoperative and postoperative IOP, ECD and pupil diameter were analyzed by paired *t*-test. The statistically significance was set when *p*-value ≤ 0.05.

## 3 Results

Nine eyes of nine patients (9 males) were included in this study. The mean age of patients was 18.9 ± 18.1 years at the time of the surgery. All patients had traumatic mydriasis and underwent lens extraction due to trauma-induced conditions including cataract, lens dislocation, or retinal detachment. The mean follow-up time was 6.3 ± 6.1 months. Baseline characteristics of the patients are provided in Table 1. Detailed comparisons of preoperative and postoperative clinical data including BCVA, IOP, ECD and pupil diameter are presented in Table 2. Table 3 shows BCVA improved from 0.94 ± 0.75 logMAR to 0.75 ± 0.67 logMAR after the surgery (*p* = 0.042), IOP decreased from 17.81 ± 6.37 mmHg to 13.70 ± 3.64 mmHg (*p* = 0.046) and pupil diameter reduced from 8.851 ± 0.686 mm to 5.263 ± 1.361 (*p* = 0.0001). The difference of ECD was not statistically significant postoperatively (*p* = 0.698). The mean spherical equivalent was −1.65 ± 3.72 D and the mean astigmatism was −2.10 ± 1.60 D among the 5 patients with available data (Supplementary Table S1). Symptoms of photophobia were graded as complete resolution, partial relief and no obvious improvement. Photophobia fully resolved in 5 patients, while 4 cases achieved partial relief but still had mild residual discomfort. On slit-lamp examination and anterior segment photographs, no clinically significant IOL decentration or gross tilt was observed in any patient throughout the follow-up period. Figure 3 shows a representative case at one month postoperatively. The slit-lamp photograph demonstrates a nearly round pupil, and the anterior segment OCT confirms the well-positioned IOL. No complications were observed till the last follow-up visit, as detailed below.

**Table 1:** Baseline clinical characteristics.

Variables	All patients (*N* = 9)
Gender	
Male: Female	9: 0
Age (years)	
MD ± SD	18.9 ± 18.1
Range	2–57
Laterality	
Right: Left	6: 3
Follow-up time (months)	
MD ± SD	6.3 ± 6.1

**Table 2:** Baseline and final clinical data before and after surgery.

Case	BCVA, logMAR		IOP, mmHg		ECD, cells/mm^2^		Pupil diameter (mm)		Postoperative complications
	Pre	Post	Pre	Post	Pre	Post	Pre	Post	
1	0.3	0.1	Tn	12.9	unmeasurable	2,359	7.66	4.55	None
2	0.4	0.4	20.8	16.7	2,819	2,885	9.43	4.37	None
3	0.5	0.3	29.8	20.5	2,700	2,658	9.21	3.33	None
4	0.52	0.52	13.8	14.9	2,473	2,468	9.07	5.96	None
5	1.7	1.7	14.2	12.1	unmeasurable	2,054	9.52	6.69	None
6	1.9	1.8	11.0	9.3	unmeasurable	1,649	7.72	3.44	None
7	0.3	0.22	14.8	13.1	2,518	2,507	9.12	6.54	None
8	uncooperative	uncooperative	Tn	Tn	unmeasurable	uncooperative	8.89	5.82	None
9	1.9	0.92	20.3	10.1	2,314	2,263	9.04	6.67	None

BCVA, best-corrected visual acuity; IOP, intraocular pressure; ECD, endothelial cell density;.

**Table 3:** Comparison of preoperative and postoperative clinical data.

Variable	Value
Preoperative BCVA (logMAR)	
MD ± SD	0.94 ± 0.75
M (IQR)	0.51 (1.53)
Postoperative BCVA (logMAR)	
MD ± SD	0.75 ± 0.67
M (IQR)	0.46 (1.26)
*p*	0.042*
Preoperative IOP (mmHg)	
MD ± SD	17.81 ± 6.37
Postoperative IOP (mmHg)	
MD ± SD	13.70 ± 3.64
*p*	0.046^†^
Preoperative ECD (cells/mm^2^)	
MD ± SD	2,564.8 ± 197.7
Preoperative ECD (cells/mm^2^)	
MD ± SD	2,556.2 ± 231.6
*p*	0.698^†^
Preoperative pupil diameter (mm)	
MD ± SD	8.851 ± 0.686
Postoperative pupil diameter (mm)	
MD ± SD	5.263 ± 1.361
*p*	0.0001^†^

*Wilcoxon signed-rank test for the analysis of BCVA.

^†^Paired t-test for the analysis of IOP, ECD and pupil diameter.

*p* < 0.05 is marked in bold.

BCVA, best-corrected visual acuity; IOP, intraocular pressure; ECD, endothelial cell density.

**Figure 3:**
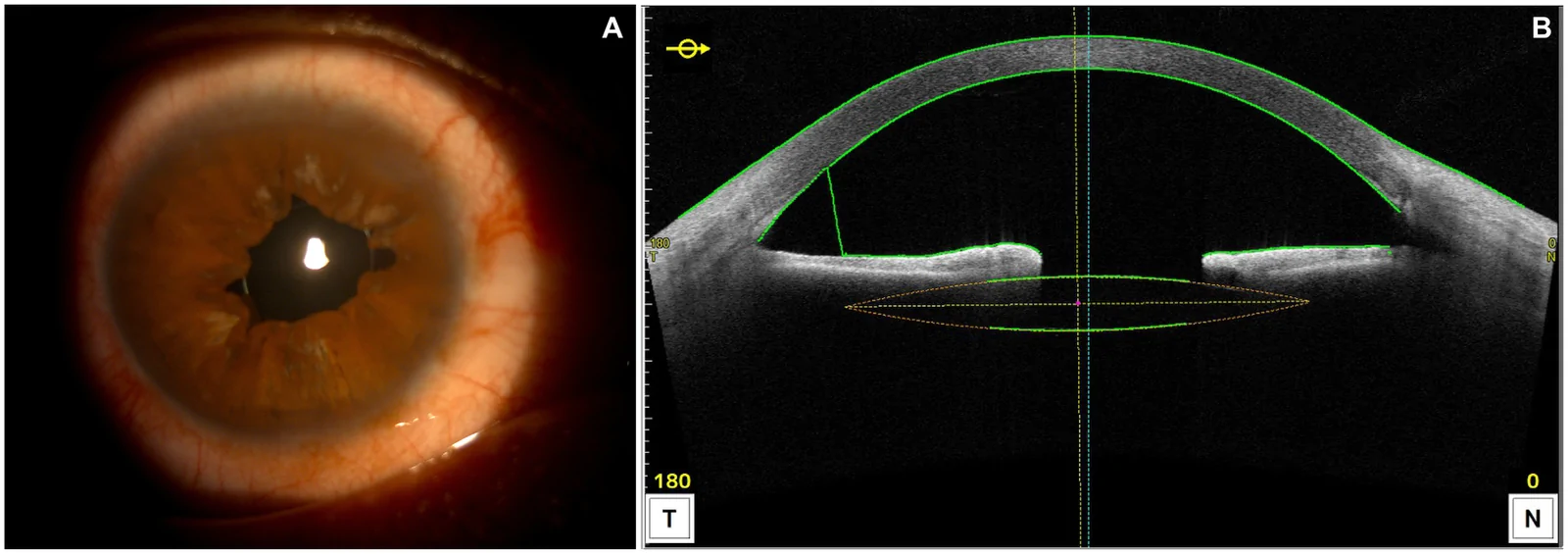
**(A)** the anterior segment photographs at 1 month postoperatively showed a round pupil. **(B)** Anterior segment optical coherence tomography revealed that the intraocular lens was centrally positioned.

A comprehensive range of potential complications was systematically assessed, including intraoperative (e.g., iris trauma, hyphema, vitreous hemorrhage, or retinal injury), early postoperative (e.g., elevated intraocular pressure, uveitis, corneal edema, hyphema, or wound leakage), and late postoperative (e.g., IOL dislocation, suture breakage or loosening, pupillary capture, cystoid macular edema, glaucoma, corneal decompensation, endophthalmitis, and retinal detachment). None of these complications were observed in any patient at the last follow-up.

## 4 Discussion

With the development of ophthalmic surgical techniques, the treatment goal for ocular trauma has evolved from merely restoring ocular anatomical structures to striving for better visual function. Studies have shown that a large pupil size would expose the junction of the optic and the haptics of an IOL, thus producing unwanted glare images (12). Meanwhile, persistent mydriasis can also cause symptoms as photophobia, diplopia, and glare, which exacerbates the impairment of visual quality (2). Therefore, it is essential to reconstruct the damaged pupil in aphakic eyes with mydriasis, which can reduce patients' subjective discomfort, stabilize the IOL, and prevent IOL capture as well as secondary glaucoma. The iris cerclage is a continuous suture technique along the pupillary margin to adjust the pupil diameter by tightening the two ends of the suture, which has been successful in treating traumatic mydriasis with good functional and cosmetic results (13, 14).

Lens capsular rupture is highly prevalent among patients with ocular trauma, which complicates the subsequent repair surgery. Scleral fixation of PCIOLs is a widely accepted surgical approach for patients with insufficient capsular support (6). Sutureless intrascleral IOL fixation combined with iris cerclage offers a promising therapeutic option, with favorable outcomes observed in clinical practice (9). While sutureless intrascleral fixation offers advantages in many cases, its application appears less suitable for pediatric patients, high myopias, or eyes with vitrectomy-related scleral scars, which may predispose to haptic dislocation or IOL tilt. In such scenarios, alternative techniques, such as scleral sutured fixation may be preferable. To the best of our knowledge, this is the first formal study in the peer-reviewed literature to report the combined use of scleral-sutured IOL fixation and iris cerclage in patients with ocular trauma. In practical application, therefore, the specific IOL fixation method needs to be selected based on the patient's actual condition.

During the follow-up period, clinical observation reported improved BCVA without severe complications in this report. Although a significant reduction in IOP was also observed, the contribution of iris cerclage to IOP reduction remains to be substantiated through large-sample-size studies. Meanwhile, ECD remained stable compared with preoperative levels during the follow-up period. In addition, this study elaborates on the optimized application of scleral-sutured IOL fixation for both three-piece (Tecnis ZA9003 and MA60AC) and single-piece IOLs (RayOne RAO600C), thereby broadening the IOL selection spectrum for eyes with traumatic aphakia. It is well established that hydrophobic foldable IOLs are the preferred choice for pediatric patients (15, 16). Compared with three-piece IOLs, single-piece hydrophobic acrylic IOLs exhibit a lower rate of posterior capsule opacification, superior stability, and better adaptability to the dynamic movements and developmental changes of the pediatric eye. Accordingly, for aphakic children with traumatic mydriasis and insufficient capsular support, the transscleral suture fixation combined with iris cerclage technique for single-piece hydrophobic IOLs proposed in this study holds important clinical significance.

Ogawa's technique made three paracenteses for suturing and we made a modification for ocular trauma patients, who often present with conditions as iris coloboma or iris synechia (13). For these patients with severe iris defect (such as the case demonstrated in the surgical technique), we chose to increase the number of side incisions and iris bites in order to reduce friction and make the shape more perfect. For patients with mild iris damage, the number of side paracenteses and iris bites can be reduced depending on the iris condition. After the pupillary sphincter loses its contractile ability, the shape of the pupil depends almost entirely on the distance from the iris bites to the pupillary margin, and the recommended distance should be approximately 1 mm. In our cohort, the number of iris bites ranged from 6 to 12 per eye, and the number of side paracenteses ranged from 4 to 6. In addition, these patients can still undergo routine fundus examinations after iris cerclage surgery. Ultra-widefield Optos fundus photography is routinely applied in patients aged over 3 years in this study, as the system enables non-mydriatic imaging through pupils ≥2.0 mm. All patients achieved a postoperative pupil diameter ≥3.0 mm after surgery in our cohort, which does not interfere with routine fundus examination.

Iris cerclage was once thought to provide a permanent treatment for traumatic mydriasis (13, 14). Nevertheless, long-term risks associated with progressive weakening of prolene sutures cannot be ignored. Declined suture tension may loosen the pupillary purse-string fixation, resulting in secondary pupil dilation and compromised pupillary shape and visual outcomes. To maintain durable surgical effects, the following strategies were implemented. First, 9-0 prolene suture was utilized in this study, which delivers superior tensile strength and durability relative to 10-0 suture. Second, individualized suture tension regulation was performed as mentioned above. Lastly, consistent postoperative follow-up was conducted to lower the incidence of related complications. In addition, Ladki et al. (17) found a risk of cerclage cheese-wiring with return of mydriasis and symptoms. Thus, long-term functional and anatomic outcomes of iris cerclage combined with scleral IOL fixation are needed further exploration. In the future, the widespread application of artificial iris may offer an additional therapeutic option for eyes with traumatic mydriasis (18). Moreover, prospective studies with standardized protocols for postoperative refraction and anterior segment OCT are warranted to more comprehensively evaluate the optical performance and IOL positional stability of this combined technique. In conclusion, scleral suture-fixated IOL implantation combined with iris cerclage pupilloplasty is an effective surgical method to achieve functional and cosmetic outcomes for aphakic eyes with traumatic mydriasis. In the future, studies with larger sample sizes and extended follow-up periods are warranted to further evaluate the efficacy and safety.

## Data Availability

The raw data supporting the conclusions of this article will be made available by the authors, without undue reservation.

## Supplementary material

Supplementary Video 1 shows one‑piece intraocular lens implantation with pupil cerclage sutureo.

Supplementary Video 2 shows the three‑piece counterpart.
