# Supplementary material for: Combined scleral suture-fixated intraocular lens implantation and Iris cerclage pupilloplasty for aphakic eyes with traumatic mydriasis
Source: Front Med (Lausanne). 2026 Sep 29;13:1958055. doi: 10.3389/fmed.2026.1958055 (PMC13657979; doi:10.3389/fmed.2026.1958055)
Supplement: Supplementary file 1 [file Supplementaryfile1.docx]

Supplementary Material

# 1 Supplementary Table 1

| **Case** | **Spherical Equivalent (SE, D)** | **Astigmatism (D)** |
| --- | --- | --- |
| **1** | −8.00 | −4.00 × 25° |
| **2** | +2.38 | −0.75 × 150° |
| **3** | −1.50 | −3.00 × 175° |
| **5** | −0.50 | 0 |
| **8** | −0.63 | −2.75 × 155° |
| **Mean ± SD** | −1.65 ± 3.72 | −2.10 ± 1.60 |
